# Serum Level of Anti-Nucleocapsid, but Not Anti-Spike Antibody, Is Associated with Improvement of Long COVID Symptoms

**DOI:** 10.3390/vaccines10020165

**Published:** 2022-01-21

**Authors:** Reka Varnai, Tihamer Molnar, Laszlo Zavori, Margit Tőkés-Füzesi, Zsolt Illes, Andrea Kanizsai, Peter Csecsei

**Affiliations:** 1 Department of Primary Health Care, Medical School, University of Pecs, 7624 Pecs, Hungary; varnai.reka@pte.hu; 2 Department of Anaesthesiology and Intensive Care, Medical School, University of Pecs, 7624 Pecs, Hungary; 3 Salisbury NHS Foundation Trust, Salisbury SP28BJ, UK; laszlo.zavori@nhs.net; 4 Department of Laboratory Medicine, Medical School, University of Pecs, 7624 Pecs, Hungary; margit.tokes-fuzesi@kk.pte.hu; 5 Department of Neurology, Odense University Hospital, University of Southern Denmark, 5230 Odense, Denmark; zsolt.illes@rsyd.dk; 6 Department of Dentistry, Medical School, University of Pecs, 7624 Pecs, Hungary; kanizsai.andrea@pte.hu; 7 Department of Neurosurgery, Medical School, University of Pecs, 7624 Pecs, Hungary; csecsei.peter@pte.hu

**Keywords:** long COVID, fatigue, spike, nucleocapsid, vaccination

## Abstract

Background: Long COVID is a condition characterized by long-term sequelae persisting after the typical convalescence period of COVID-19. Previous reports have suggested the role of an unsatisfactory immune response and impaired viral clearance in the pathogenesis of long COVID syndrome. We focused on potential associations between post-vaccination changes of antibody titers and the severity of long COVID symptoms and factors influencing the state of remission observed in patients with long COVID after vaccination. Methods: The severity of long COVID symptoms and serum anti-SARS-CoV-2 spike (S-Ig) and nucleocapsid (NC-Ig) levels were assessed in 107 post-COVID subjects at two time points: at baseline, and 17–24 weeks later. Besides, vaccination status was also assessed. Symptoms were evaluated based on the Chalder fatigue scale (CFQ-11) and visual analogue scale (VAS). Results: Serum level of S-Ig and NC-Ig at baseline were significantly higher in the patients with non-severe fatigue than those with severe fatigue, and this difference remained significant at follow-up in the case of NC-Ig. NC-Ig level above median was as an independent predictor for complete remission at follow-up. The difference in NC-Ig levels in subgroup analyses (severe fatigue vs. non-severe fatigue; complete remission vs. incomplete remission or progression) was found to be significant only in patients who received vaccination. Conclusions: The immune response against the SARS-CoV-2 nucleocapsid may play a more important role than the spike in the course of long-term COVID syndrome.

## 1. Introduction

By 3 December 2021, there were more than 260 million confirmed cases of COVID-19, including 5,233,000 deaths, reported to WHO [1]. Most patients with COVID-19 return to baseline health status after acute infection with SARS-CoV-2, but a significant proportion report ongoing health issues. In a prospective cohort study from China, a majority of formerly hospitalized patients with COVID-19 reported at least one symptom 6 months after onset of the disease, mainly troubled with fatigue or muscle weakness, sleep difficulties, and anxiety or depression [2]. In the United Kingdom, the National Institute for Health and Care Excellence (NICE) defined long COVID as signs and symptoms developing during or after COVID-19 infection and persisting for more than 12 weeks that are not explained by an alternative diagnosis [3]. To date, management of patients with long COVID is based solely on general advice, supported self-management, and multidisciplinary rehabilitation [3]. Previously, our team assessed the correlation between the severity of post-COVID symptoms in an unvaccinated cohort of patients and their serum SARS-CoV-2 antibody levels [4]. This prospective study revealed that those with severe fatigue had significantly lower anti-spike and anti-nucleocapsid titers, regardless of the time elapsed from the onset of the disease. We hypothesized that chronic inflammation might contribute to the symptoms of long-standing fatigue and that the noticeably lower antibody levels are due to an unsatisfactory immune response to the virus. Our study concluded that a pronounced immune response triggered by the SARS-CoV-2 vaccines might facilitate the elimination of viral antigen residues, which consequently might improve the symptoms of patients suffering from long COVID. In accordance with this, *Tran* et al. recently reported that vaccination reduced the severity of symptoms of long COVID and doubled the remission rate of such symptoms at 120 days after vaccination [5]. The primary objective of this follow-up study was to explore factors influencing the dynamics of fatigue and potential associations between post-vaccination changes of antibody titers and fatigue status. Our secondary objective was to investigate which factors influence the complete remission state observed at the follow-up visit.

## 2. Methods

### 2.1. Study Population

All patients with SARS-CoV-2 infection treated for acute COVID-19 as out- or inpatients at the 1st Department of Internal Medicine of the University of Pecs, with symptom onset between October 2020 and May 2021, were enrolled after written informed consent into this follow-up study. Importantly, at the time of recruitment all participants were presented beyond 30 days after symptom onset. In this study, the long-term course of post-COVID symptoms, including fatigue and antibody status of each subject, were analyzed at baseline and at follow-up. Patients seeking GP assistance with post-COVID related symptoms were followed up in an outpatient clinic, where they had their baseline parameters documented and their eligibility for the study established.

After routine safety studies including blood pressure, ECG recording, and blood sampling, patients completed the validated Chalder fatigue scale [6]. Based on this, participants were dichotomized into severe fatigue vs. non-severe fatigue subgroups (see fatigue and symptom severity assessment, outcomes). Antibody titers and baseline laboratory testing were processed by the Department of Laboratory Medicine in a blinded manner to patient data. The inclusion criteria were the following: patients needed to be older than 18 years old, they had to be symptomatic at the time of their presentation to the clinic, they needed to have at least one positive antigen- or PCR test, and at least 30 days needed to elapse between their presentation and the onset of symptoms. Patients with pre-existing malignancies or autoimmune conditions, those on immunosuppressive treatment, those with acute coronary syndrome, and those who had previously received a SARS-CoV-2 vaccine or had any condition that might significantly interfere with the assessment of fatigue were excluded from the study.

Patient-related data were collated from electronic healthcare records, including the relevant details of hospitalization, the exact onset-time of symptoms, the need for oxygen treatment, antiviral medication (remdesivir, favipiravir), and date and type of vaccination. Participants were also interviewed for demographic and index disease related information.

All participants had a follow-up assessment approximately 17–24 weeks after baseline, when evaluation of fatigue and vaccination status was repeated. Participants were only asked to confirm vaccination status after fatigue assessment to minimize bias due to a perceived association between the assessment and vaccination. Serum-IgG antibodies against SARS-CoV-2 were determined again by the same unit as at the baseline visit using the same protocol for antibody analysis.

### 2.2. Fatigue and Symptom Severity Assessment, Outcomes

We worked with an official translation agency to translate the Chalder-fatigue scale (CFQ-11) into Hungarian and used it to assess the participant’s level of fatigue. We used the Likert scoring system with a range of 0–3 and calculated the subscale scores for physical (0–21) and psychological fatigue (0–12) with a final global score of 0–33 [7].

We used a bimodal score ranging from 0–11 to create the case definition of severe fatigue as the primary endpoint [7,8]. To establish the final score, we used 0 if the patient selected “less than usual” or “no more than usual” and assigned 1 if they chose “more than usual” or “much more than usual”. We determined the case definition of severe fatigue as a final score of 4 or more [7,8].

In addition, all patient complaints during the post-COVID period were recorded by qualified independent physicians in a face-to-face interview. Patient-reported complaints were assessed as existing only if they were present up to 7 days prior to the follow-up visit. To evaluate the level of being affected by the symptoms, participants were asked to score each symptom from 0 (have no problem) to 10 (have extreme) on a visual analog scale (VAS). Patients were asked to rate their symptoms and the sum of these formed the total of visual analogue scale (VAS) value indicating the overall severity of symptoms. The median value of VAS was calculated from the total VAS value registered at follow-up visit. As a secondary endpoint, we have investigated the disease remission rate. The definition of complete remission was as follows: bimodal score = 0 and VAS scale = 0, both calculated at follow-up visit.

### 2.3. Laboratory Analysis and Assay

We collected peripheral venous blood samples, immediately centrifuged them at 3500 r/min for 15 min, and stored the supernatant at −80 °C until further analysis. We used a fully automated Cobas e801 analyzer from Roche Diagnostics to detect SARS-CoV-2 antibodies. The quantitative analysis of anti-SARS-CoV-2 spike proteins was performed by using the Elecsys^®^Anti-SARS-CoV-2 S assay (an electrochemiluminescence immunoassay by Roche Diagnostics, Basel, Switzerland). This specific assay detects high-affinity SARS-CoV-2 antibodies using a recombinant protein mimicking the receptor binding domain (RBD) of the spike (S) protein in a double-antigen sandwich assay format. To detect the antibodies against the nucleocapsid protein in a qualitative manner, we used the Elecsys^®^Anti-SARS-CoV-2 electrochemiluminescence immunoassay by Roche Diagnostics that uses a recombinant protein mimicking the nucleocapsid (NC) antigen in a double-antigen sandwich assay format. Although the test predominantly captures anti-SARS-CoV-2 IgG immunoglobulins (Ig), it also binds to anti-SARS-CoV-2 IgA and IgM and includes both biotinylated and ruthenylated forms of SARS-CoV-2 recombinant nucleocapsid and spike antigens. By utilizing streptavidin -coated microparticles, the complex can be magnetically captured following binding to the solid phase via a biotin-streptavidin reaction. Results were reported as numeric values in the form of a cut-off index (COI; signal sample/cutoff) for the anti-NC antibodies, which should be reported as well as in the form of a qualitative results non-reactive (COI < 1.0; negative) and reactive (COI ≥ 1.0; positive). We used a cut-off value < 0.8 U/mL for the anti-S antibodies and a cut-off value of <1 U/mL for the anti-NC antibodies.

### 2.4. Ethical Approval

The Medical Research Council of Hungary (IV/2505-3/2021/EKU) approved the study. We followed the ethical guidelines of the 1975 Declaration of Helsinki during all procedures and obtained written informed consent from all participants before their enrollment into the research.

### 2.5. Statistics

We used SPSS (version 26; IBM, Armonk, NY, USA) software to evaluate statistical data. We used the Kolmogorov–Smirnov test to check for normality, the chi-square test for categorical variables, and the Student-t test for quantitative data of demographic and clinical factors. We compared non-normally distributed data with the Mann–Whitney test and presented them as median and interquartile range (25th–75th percentiles). We calculated the Spearman’s correlation coefficient (rho) for correlation analysis and used ROC analysis to determine the best cut-off value of predictors. We considered a *p*-Value < 0.05 as statistically significant.

## 3. Results

### 3.1. Characteristics

Of the 232 patients who were screened and assessed for eligibility, 139 patients were enrolled in the study. A total of 93 patients were excluded because they did not fulfill inclusion criteria at baseline visit (see Supplementary Figure S1). A total of 32 patients refused to attend for follow-up assessment. Hence, 107 patients completed the study. The mean age of the patients was 50.2 ± 12 years; 66 patients (61.7%) were female, and 40 patients (37.4%) were hospitalized during acute COVID-19 infection. Thirty-seven patients (34.6%) received antiviral medication. The median length between symptom onset of the disease and the baseline visit was 65 (IQR, 46–99) days, the median length of symptom onset to follow-up visit was 207 (IQR, 179–241) days, and the median interval between baseline and follow-up visit was 143 (IQR, 119–170) days. Eighty-four patients (78.5%) have received second-dose of COVID-19 vaccine. The median interval between first-dose vaccine and the follow-up visit was 96 days (IQR, 48–126). Seventy-five percent of all vaccines were mRNA-based vaccines (BNT162b2 and mRNA-1273). The vaccination schemes were homologous in all cases.

### 3.2. Fatigue Status and Antibody Level

**Figure 1:**
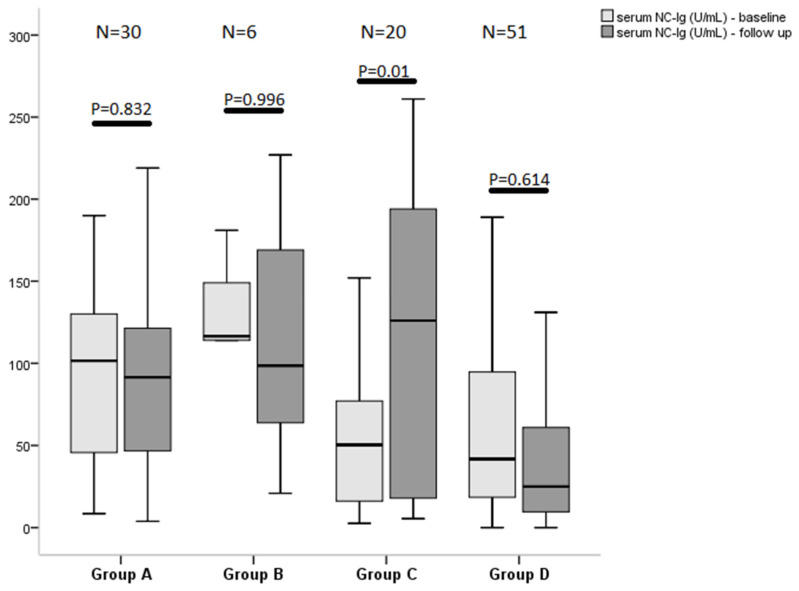
Association between the severity of post-COVID fatigue and serum level of anti-SARS-CoV-nucleocapsid immuneglobulins at baseline and follow-up visits. We used the Chalder-fatigue scale (CFQ-11) to establish a case definition for severe fatigue by utilizing a bimodal scoring system ranging between 0–11. A case of severe fatigue was defined as a score of 4 or more. Group A, (N = 30): patients experienced severe fatigue neither at baseline nor at follow-up (bimodal score < 4 at both visit). Group B, (N = 6): patients only had severe fatigue at follow-up (bimodal score ≥ 4), and baseline bimodal score was <4. Group C, (N = 20): patients had severe fatigue (bimodal score ≥ 4) at the baseline visit but not at follow-up. Group D, (N = 51): at both visits, patients experienced severe fatigue (bimodal score ≥ 4). Time of baseline visit was at least 30 days after COVID-19 symptom onset. Next, follow-up visit was 17–24 post-baseline weeks. Nucleocapsid IgA + IgM + IgG level (NC-Ig). Statistical analysis was performed using Mann–Whitney-U test in each group, respectively.

**Figure 2:**
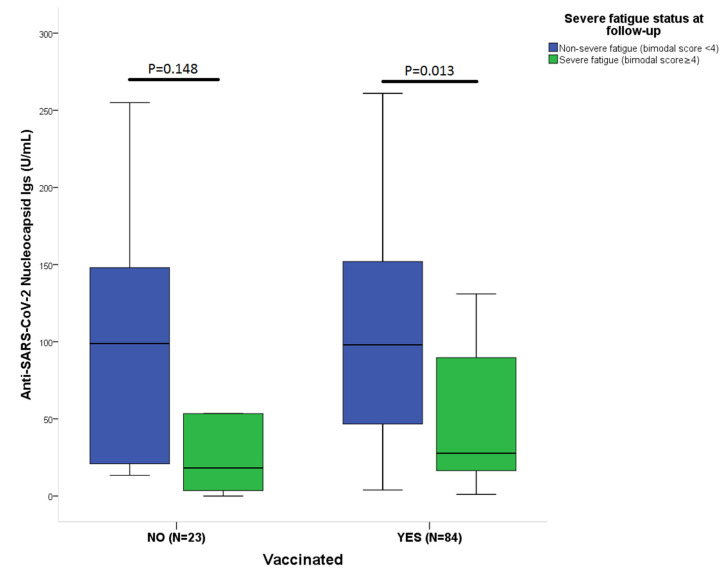
Patients with severe fatigue show decreased level of serum antiSARS-CoV-2 nucleocapsid-Ig (A + M + G) antibody levels both in unvaccinated (non-severe fatigue, N = 9 vs. severe fatigue, and N = 14) and vaccinated (non-severe fatigue, N = 41 vs. severe fatigue, and N = 43) individuals but significance is observed only in the vaccinated group. The case definition of severe fatigue was established using a combination of a bimodal scoring system and the CFQ-11 scale, resulting in a final score ranging between 0–11. A score of 4 or more was defined as severe fatigue. Statistical analysis was performed using Mann–Whitney-U test.

Patient characteristics by fatigue status at baseline and on follow-up are shown in Table 1. Fatigue was assessed using the CFQ-11 in all participants: the median (IQR) score at baseline was 17 (12–20) across the study population and was 14 (8–18) at follow-up. The median physical fatigue score (IQR) was 13 (9–15) at baseline and 10 (6–13) at follow-up, while the median psychological fatigue score (IQR) was 4 (2–6) and 4 (1–6), respectively. The median serum SARS-CoV-2-nucleocapsid (NC-Ig) level for patients with severe fatigue was 34 (IQR: 14–89) for the entire study period and 100 (IQR: 43–145) for patients with non-severe fatigue (*p* < 0.001), while the median serum SARS-CoV-2-spike-Ig (S-Ig) level for patients with severe fatigue was 231 (IQR: 63–3189) and was 1182 (IQR: 174–9836) for patients with non-severe fatigue (*p* = 0.001). The serum level of both anti-S- and anti-NC-Ig was elevated in patients with non-severe fatigue compared to those with severe fatigue at baseline, but at the follow-up visit only NC-Ig was significantly different between the two fatigue groups. Univariate analysis revealed the following associations with severe fatigue status at follow-up: hospitalization due to the index infection, serum level of NC-Ig, use of antiviral medication during hospitalization, total value of post-COVID symptoms on a VAS scale, and number of post-COVID symptoms, all at baseline. In a binary logistic regression analysis, none of the above variables proved to be an independent predictor of severe fatigue. Association between the severity of post-COVID fatigue and the serum level of NC-Ig at baseline and follow-up visits is depicted at Figure 1. There was a significant difference in serum NC-Ig values between the two fatigue groups in those patients, who were vaccinated before the follow-up visit, Figure 2.

### 3.3. Symptoms Severity at Follow-Up and Antibody Level

During the follow-up, the median value of total number of post-COVID symptoms was 4 (IQR, 1–7). The median value of VAS was 16.5 (IQR, 1–32; min: 0, max: 185), and 32 patients (30%) had a complete remission. A binary logistic regression analysis revealed an independent association at follow-up between the level of serum NC-Ig and the total VAS score (OR = 0.986; 95% CI: 0.977–0.994; *p* = 0.001) entering the model confounders, including age, gender, interval between symptom onset and follow-up, need for hospitalization, antiviral medication during acute disease, and serum level of S-Ig, Table 2.

Next, a separate statistical analysis was run with the median value of anti-NC-Ig as an outcome of interest. Based on binary logistic regression analysis, the interval between symptom onset and follow-up (OR = 0.985; 95%CI: 0.972–0.999; *p* = 0.032), the mean value of VAS at follow-up (OR = 0.606; 95% CI: 0.443–0.829; *p* = 0.002), and age (OR = 1.060; 95% CI = 1.005–1.117; *p* = 0.032) proved to be independent predictors of the median NC-Ig level as a cutoff. Univariate analysis found that the need for hospitalization during the acute disease, antiviral medication, and the median NC-Ig value at follow-up were associated with complete remission (bimodal score = 0, VAS score = 0) at follow-up. Importantly, the binary logistic regression analysis also indicated that only the median NC-Ig level at follow-up (OR = 0.337; 95% CI = 0.120–0.946; *p* = 0.039) was a significant independent contributor of complete remission status at follow-up, Table 2.

### 3.4. Effect of Vaccination on Antibody Levels and Outcome

In vaccinated patients (N = 84), the median serum NC-Ig level for patients with complete remission at follow-up (bimodal score = 4, VAS scale = 0) was significantly higher than in patients with incomplete remission or progression (median: 100, IQR: 50–158 vs. 32, IQR: 16–94; *p* = 0.024). Patients with severe fatigue in the vaccinated group had significantly lower level of serum NC-Ig than patients with non-severe fatigue (28, 16–94 vs. 97, 38–155; *p* = 0.022). In unvaccinated patients, this difference was not significant in regard either the fatigue or the complete remission outcome measures. There was also no difference in serum S-Ig levels between the vaccinated and non-vaccinated groups for these endpoints.

## 4. Discussion

Our studies show three critical findings: firstly, serum level of S-Ig and NC-Ig at baseline were significantly higher in the patients with non-severe fatigue than those with severe fatigue, and this difference remained significant at follow-up only in the case of NC-Ig. Secondly, the logistic regression analysis identified serum NC-Ig level > median as an independent predictor for complete remission status at follow-up. Thirdly, in the group that showed clinical improvement at the follow-up visit, a significantly higher NC-Ig level was observed compared to the other groups (unchanged or worsened fatigue status at follow-up compared to baseline). Finally, the difference in NC-Ig levels in the examined subgroups (severe fatigue vs. non-severe fatigue and complete remission vs. incomplete remission or progression) was significant only in patients who received vaccination.

A recent study found a clear association between improvement in long-term COVID symptoms and vaccination [5]. According to the hypothesis explaining these findings, which was also formulated by the present authors in an earlier article [4], the disease is mediated by a persistent viral reservoir and/or by circulating virus fragments, and COVID-19 vaccine may promote and activate the entire immune system to eliminate these remaining viral antigens and lead to the improvement of symptoms. Another survey of 900 patients with long COVID conducted by an advocacy group in the UK [9] reported that 56.7% of participants perceived an improvement in their symptoms after their first COVID-19 vaccine injection; however, the underlying exact mechanism is not yet clear.

In general, one of the main theories is the insufficient clearance of viral fragments. The patients who have low levels of antibodies at the time of hospital discharge may have a high risk of developing redetectable SARS-CoV-2 RNA on RT-PCR testing after recovery. These findings indicate the important role of these antibodies in viral clearance [10]. SARS-CoV2 nucleocapsid protein has RNA-binding and chaperone activities and promotes SARS-CoV-2 gRNA replication [11]; thus, an inadequate humoral immune response (against nucleocapsid antigen) may indirectly promote further viral replication and lead to further partial persistence of symptoms. Other evidence also supports this assumption. SARS-CoV-2 nucleic acid is detectable long after the resolution of symptoms in a significant percentage of previously diagnosed individuals; however, it is unclear whether persistent PCR positivity by NP swab is due to persistent infection with transmissible virus or nontransmissible nucleic acid fragments [12]. In an animal study, that vaccination with a human adenovirus type 5 vector expressing the SARS-CoV-2 nucleocapsid protein can establish protective immunity, to broaden epitope coverage and immune effector mechanisms in mice and this vaccine against nucleocapsid antigen elicits spike-independent SARS-CoV-2 protective immunity [13]. Taking into account the evidence discussed and our results, high NC-Ig levels in patients with better outcomes highlight the importance of humoral immunity against NC antigen.

Another important factor in the association between antibody levels and long COVID syndrome may be the presence of cross-reactive antibodies to previous coronavirus infection. Immune cross-reactivity among seasonally spreading human coronaviruses (HCoVs) has long been hypothesized to provide effective but transient cross-protection against distinct HCoVs [14]. SARS-CoV-2 spike glycoprotein (S)-reactive antibodies were detectable using a flow cytometry-based method in SARS-CoV-2-uninfected individuals and were particularly prevalent in children and adolescents [14]. Exposure of the population to other coronaviruses prior to the COVID-19 pandemic resulted in some degree of cross-protection against SARS-CoV-2 infection [15]. Nucleocapsid proteins from HCoV-NL63 and HCoV-229E were detected in most samples, thereby implicating prior exposure to these two HCoVs as the likely source of cross-reactive antibodies against SARS-CoV-2 [15]. The presence of antibodies to other types of coronaviruses, particularly against nucleocapsid antigen, may affect the severity of persistent symptoms after SARS-CoV-2 infection and may contribute to the higher level in patients with milder symptoms.

Chronic fatigue syndrome (CFS) is frequently preceded by a viral infection [16], but the molecular mechanisms underlying these post-acute presentations have yet to be elucidated. Post-infectious sequelae have also been observed following infection by other coronaviruses such as SARS-CoV and MERS-CoV; the most common symptom is fatigue [17]. Cytotoxic activity of NK and CD8+ T cells and NK phenotypes was significantly decreased in CFS patients, suggesting significant dysregulation of the immune system in CFS patients [18]. In patients with CFS, the CD8+ T cells had reduced mitochondrial membrane potential compared with those from healthy controls and CD8+ T cells had reduced glycolysis following activation [19]. These findings indicate that patients have impaired T cell metabolism. The T cell compartment was altered in CFS population, with increased proportions of effector memory CD8+ T cells and decreased proportions of terminally differentiated effector CD8+ T cells reflecting an altered immunological state caused by an ongoing or recent infection [20]. There is considerable evidence for dysfunction of the CD4+ and especially CD8+ T cells in chronic fatigue syndrome, and given the marked similarity to the symptoms of SARS-CoV-2-induced post COVID fatigue, the pathophysiological role of these T cells in long COVID also arises.

A longitudinal follow-up of 254 COVID-19 patients revealed that not only did SARS-CoV-2 specific CD8+ T cells show a pronounced preference to recognize the NC protein but also that the CD8+ T cell response rate was primarily directed to the nucleocapsid protein (57%). During a natural occurring infection, the antigen-presenting cells might display a higher proportion of NC-protein-derived peptides, leading to a higher number of nucleocapsid-specific CD8+ T cells [21].

Based on these results, it is likely that vaccine induction of CD8+ T cells to more conserved antigens such as the nucleocapsid, rather than just to SARS-CoV-2 spike antigens, may add benefit to more rapid containment of infection [21]. Patients with long COVID had reduced CD4+ and CD8+ effector memory (EM) cell numbers; this T cell perturbations persisted for several months after mild COVID-19 and was associated with long COVID symptoms [22]. Seeing the importance of the CD8+ cells against nucleocapsid in an adequate immune response against SARS-CoV-2, it is hypothesized that the inadequacy of humoral immunity, i.e., the low titer of antibodies against nucleocapsid, also draws attention to the inadequate immune response and consequently may be a cause of persistent symptoms after COVID-19. However, the presence of antibodies does not always predict the presence of specific T cells or memory B cells [23].

The limitations of our study include a relatively small sample size and the fact that fatigue is often difficult to measure in an objective manner due to its inherently subjective nature. We did not look for potential baseline structural organic abnormalities in our patients with post-COVID symptoms, and although cellular immunity plays an important role in the immune response against SARS-CoV-2, we decided not to examine this process in our research. We also recognize that despite the fact the English version of the CFQ-11 scale is formally validated, an official translation of the scale does not necessarily account for cross-cultural adaptation and does not guarantee validity. No conclusions concerning the NC-Ig levels should be drawn for the non-vaccinated group due to the low sample size.

## 5. Conclusions

The serum level of both S-Ig and NC-Ig was elevated in patients with non-severe fatigue compared to those with severe fatigue at baseline, but the difference remained significant only in the case of NC-Ig at the follow-up visit. Nevertheless, NC-Ig did not prove to be an independent predictor of severe fatigue. Importantly, the serum level of NC-Ig higher than its median value was observed as an independent predictor for complete remission status at follow-up. In addition, the difference in NC-Ig levels in the examined subgroups (severe fatigue vs. non-severe fatigue and complete remission vs. incomplete remission or progression) was significant only in patients who received vaccination. These results may suggest that the immune response against the nucleocapsid antigen (humoral/cellular) is an important factor in the improvement of long COVID symptoms, and these findings may also help to reduce vaccine reluctancy and scepticism among patients suffering from long COVID.

## Figures and Tables

**Table 1:** Patient characteristics by fatigue status at baseline and at follow-up.

		Baseline (N = 107)			Follow-Up (N = 107)		
		Non-Severe Fatigue	Severe Fatigue		Non-Severe Fatigue	Severe Fatigue	
Characteristics		N = 36 (33.6%)	N = 71 (66.4%)	*p* Value	N = 50 (46.7%)	N = 57 (53.3%)	*p* Value
Female	N, %	16 (44)	50 (70)	0.009	27 (54)	39 (68)	0.126
Age	Years (mean ± SD)	53 ± 12	49 ± 11	0.035	50 ± 12	50 ± 12	1.000
BMI	mean ± SD	28.5 ± 8	26.5 ± 4	0.200	27.7 ± 7	26.7 ± 5	0.748
Hospitalization	N, %	14 (38.9)	26 (36.6)	0.819	25 (50)	15 (26.3)	0.012
O_2_-supplementation	N, %	5 (13.9)	14 (19.7)	0.456	12 (24)	7 (12)	0.113
Antiviral medication	N, %	13 (36.1)	24 (33.8)	0.813	25 (50)	12 (21)	0.002
Vaccinated	N, %	N/A *	N/A *	N/A *	41 (82)	43 (75.4)	0.410
mRNA-based	N, %	N/A *	N/A *	N/A *	30 (73)	33 (77)	0.658
Vector-based	N, %	N/A *	N/A *	N/A *	6 (15)	8 (19)	0.522
Inactivated	N, %	N/A *	N/A *	N/A *	5 (12)	2 (4)	0.08
Time from vaccination (1.dose) to follow-up in days	days, mean ± SD	N/A *	N/A *	N/A *	98 (56–134)	90 (48–122)	0.495
Total CFQ-11 Score (Liekert Scoring)	mean ± SD	11 (7–13)	19 (17–22)	<0.001	8 (3–11)	17 (15–21)	<0.001
Physical Fatigue (CFQ-11 items 1–7)	mean ± SD	7 (6–9)	14 (13–16)	<0.001	6 (3–7)	13 (11–14)	<0.001
Psychological Fatigue (CFQ-11 items 8–11)	mean ± SD	4 (1–4)	5 (4–7)	<0.001	2 (0–4)	5 (4–7)	<0.001
Total CFQ-11 Score (Bimodal Scoring)	mean ± SD	1 (0–3)	7 (6–8)	<0.001	0 (0–2)	6 (5–8)	<0.001
anti-SARS-CoV-2 S-Ig	U/mL, median, IQR	183 (106–696)	113 (28–246)	0.003	6949 (1430–12,500)	3723 (911–10,932)	0.155
anti-SARS-CoV-2 NC-Ig	U/mL, median, IQR	104 (48–131)	45 (18–89)	<0.001	98 (29–152)	27 (10–85)	0.002
Symptom onset to baseline	day, median, IQR	74 (56–100)	60 (40–99)	0.126	65 (42–93)	69 (46–103)	0.359
Symptom onset to follow-up	day, median, IQR	N/A *	N/A *	N/A *	203 (179–233)	208 (179–256)	0.512
Interval baseline to follow-up	day, median, IQR	N/A *	N/A *	N/A *	142 (119–171)	148 (119–168)	0.837

The categorical variables are displayed presented as frequency (%), and the continuous variables are displayed presented as mean ± standard deviation (SD) or median with interquartile range (IQR). We used chi-square, Fisher exact test, Student’s *t*-test, or Mann–Whitney U test as appropriate to assess inter-group differences between the case-group defined by our case definition of severe fatigue using the CFQ-11 scale and the control group. The inter-group differences were assessed using chi-square test, Fisher exact test, Student’s *t*-test, or Mann–Whitney U test as appropriate in order to compare differences in those without non-severe fatigue and those with severe fatigue as per the CFQ-11 “caseness” definition for severe fatigue. Abbreviations: N, number; BMI, body mass index; VAS, visual analogue scale; CFQ-11, Chalder fatigue scale; COVID, coronavirus disease; SARS, severe acute respiratory syndrome; S-Ig, spike immunoglobulin; and NC-Ig, nucleocapsid immunoglobulin. * N/A: Not Applicable.

**Table 2:** Binary logistic regression analysis assessing correlations between anti-SARS-CoV-2 nucleocapsid antibody level, complete remission status at follow-up, mild symptoms at follow-up (bimodal score ≠ 0; mean Total VAS score 0–2), and demographic/clinical variable.

	Value of NC-Ig at Follow-Up (U/mL, Median as the Cutoff) ^§^			
Variables	B	Odds Ratio	95% CI	*p*-Value
Total CFQ-11 at follow-up	0.013	1.013	0.913–1.123	0.811
Hospitalization	0.441	1.555	0.114–21.152	0.740
Antiviral medication	−0.679	0.507	0.035–7.296	0.618
Interval between symptom onset and follow-up	−0.015	0.985	0.972–0.999	0.032
Mean value of VAS at follow-up	−0.501	0.606	0.443–0.829	0.002
Age	0.058	1.060	1.005–1.117	0.032
Total number of symptoms at follow-up	−0.053	0.949	0.767–1.172	0.625
Gender	−0.602	0.547	0.163–1.840	0.330
	Complete remission at follow-up (bimodal score = 0, VAS score = 0)			
Variables	B	Odds ratio	95% CI	*p*-Value
Hospitalization	−0.401	0.670	0.084–5.312	0.704
Antiviral medication	−0.546	0.579	0.071–4.746	0.611
NC median ^§^ (follow-up)	−1.089	0.337	0.120–0.946	0.039
Gender	−0.356	0.701	0.265–1.849	0.472
Age	−0.005	0.995	0.954–1.038	0.829
	Total VAS score at follow-up (median as the cutoff) ^§^			
Variables	B	Odds ratio	95% CI	*p*-Value
Age	0.012	1.012	0.968–1.058	0.599
Gender	1.110	3.034	1.063–8.656	0.038
NC-Ig (U/mL, follow-up)	−0.015	0.986	0.977–0.994	0.001
Interval between symptom onset and follow-up	0.007	1.007	0.996–1.019	0.210
Antiviral medication	1.224	3.401	0.392–29.492	0.267
Hospitalization	−0.352	0.704	0.088–5.616	0.740
S-Ig (U/mL, follow-up)	0.000	1.000	1.000–1.000	0.882

^§^ In the binary logistic regression models displayed above, we assigned a binary dependent variable to serum antibody levels and VAS based on their median value (0: ≤median, 1: >median). In these binary logistic regression models, serum antibody levels and VAS score were converted to a binary dependent variable, based on the median value of the sample (0: ≤median, 1: >median). Abbreviations: NC-Ig, nucleocapsid immunglobulin A + M + G); S-Ig, spike immunglobulin A + M + G; VAS, visual analogue scale; CFQ-11, Chalder Fatigue Scale; COVID, coronavirus disease; and SARS, severe acute respiratory syndrome.

## Data Availability

All relevant data are within the manuscript.

## Supplementary Materials

The following supporting information can be downloaded at: https://www.mdpi.com/article/10.3390/vaccines10020165/s1, Figure S1: Flowchart of participants.
