# Supplementary figures and images for: Serum Level of Anti-Nucleocapsid, but Not Anti-Spike Antibody, Is Associated with Improvement of Long COVID Symptoms
Source: Vaccines (Basel). 2022 Jan 21;10(2):165. doi: 10.3390/vaccines10020165 (PMC8924883; doi:10.3390/vaccines10020165)

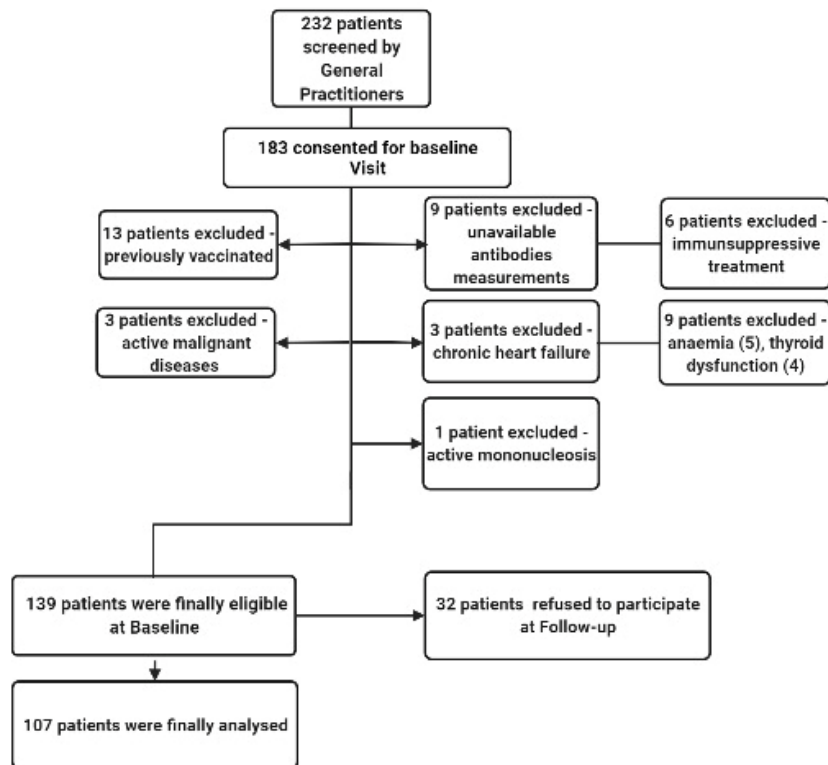

**Figure S1.** Flowchart of participants.

Supplement: Supplementary file 1 [file vaccines-10-00165-s001.zip › vaccines-1547083-SI.pdf]
